# Effects of Female Reproductive Hormone Levels on Inadvertent Intraoperative Hypothermia during Laparoscopic Gynecologic Surgery: A Retrospective Study

**DOI:** 10.3390/medicina57111255

**Published:** 2021-11-16

**Authors:** Cheol Lee, SeongNam Park, ByoungRyun Kim, Hyeonbin Yim, Myeongjong Lee, Juhwan Lee, Hyungtae Kim

**Affiliations:** 1 Department of Anesthesiology and Pain Medicine, Wonkwang University School of Medicine, 895 Muwang-ro, Iksan 54538, Korea; ironyii@wku.ac.kr (C.L.); hb3409@naver.com (H.Y.); 2 Department of Obstetrics & Gynecology, Wonkwang University School of Medicine, 895 Muwang-ro, Iksan 54538, Korea; parksn77@naver.com (S.P.); brkim74@wku.ac.kr (B.K.); 3 Department of Anesthesiology and Pain Medicine, Konkuk University Medical School, 82 Gugwondae-ro, Chungju 27376, Korea; gooddr21@kku.ac.kr; 4 Department of Anesthesiology and Pain Medicine, Asan Medical Center, University of Ulsan College of Medicine, Seoul 05505, Korea

**Keywords:** hypothermia, gynecologic surgery, reproductive hormones

## Abstract

*Background and Objectives:* Female reproductive hormones may affect core body temperature. This study aimed to investigate the effects of female reproductive hormones on inadvertent intraoperative hypothermia in patients who underwent laparoscopic gynecologic surgery under general anesthesia. *Materials and Methods:* This retrospective study included 660 menstruating and menopausal female patients aged 19–65 years. The patients were divided into two groups according to the occurrence of inadvertent intraoperative hypothermia: non-hypothermia group (*N* = 472) and hypothermia group (*N* = 188). After propensity score matching, 312 patients (*N* = 156 in each group) were analyzed to investigate the association between intraoperative hypothermia and female reproductive hormones. As potential predictors of inadvertent hypothermia, the levels of female reproductive hormones were analyzed using binary logistic regression. *Results:* The association of estradiol (r = −0.218, *p* = 0.000) and progesterone (r = −0.235, *p* = 0.000) levels with inadvertent intraoperative hypothermia was significant but weakly negative before matching; however, it was significant and moderately negative after matching (r = −0.326, *p* = 0.000 and r = −0.485, *p* = 0.000, respectively). In a binary logistic analysis, the odds ratio for estradiol was 0.995 (*p* = 0.014, 0.993 < 95% confidence interval [CI] < 0.998) before matching and 0.993 (*p* = 0.000, 0.862 < 95% CI < 0.930) after matching, and that for progesterone was 0.895 (*p* = 0.000, 0.862 < 95% CI < 0.930) before matching and 0.833 (*p* = 0.014, 0.990 < 95% CI < 0.996) after matching. *Conclusions:* Estradiol and progesterone levels were associated with inadvertent intraoperative hypothermia. However, the odds ratio for female reproductive hormone levels was close to 1. Therefore, female reproductive hormones may not be a risk factor for hypothermia during gynecologic surgery under general anesthesia. However, a small sample size in this study limits the generalizability of the results.

## 1. Introduction

The incidence of inadvertent intraoperative hypothermia is reported to be between 20% and 90%, depending on the surgical population and demographic characteristics of the patients [1,2]. Inadvertent intraoperative hypothermia is defined as a core body temperature of <36 °C in patients undergoing surgery under general anesthesia [1,2,3,4,5,6]. The consequences of intraoperative hypothermia include increased incidence of postoperative adverse events, such as surgical-site infection, pain, and prolonged recovery and hospitalization [1,2,3,4,5,6].

The mechanism by which the reproductive hormones affect thermoregulatory control during the menstrual cycle in females has been described [7,8,9,10,11]. Progesterone inhibits the activity of warm-sensitive neurons, thus increasing body temperature by inhibiting heat-loss mechanisms. In contrast, estrogen inhibits the activity of cold-sensitive neurons and stimulates the warm-sensitive neurons, thus decreasing the body temperature by inhibiting heat-retention mechanisms and activating the heat-loss mechanisms. Therefore, the phase of the menstrual cycle might affect the core body temperature under general anesthesia due to its effects on thermoregulatory control [8].

However, no clinical study has investigated the effects of the levels of female reproductive hormones on core body temperature and the risk of inadvertent intraoperative hypothermia.

We hypothesized that the levels of female reproductive hormones might influence the occurrence of inadvertent intraoperative hypothermia. This study aimed to investigate whether the levels of female reproductive hormones through the different phases of the menstrual cycle can predict inadvertent hypothermia in patients undergoing laparoscopic gynecologic surgery under general anesthesia.

## 2. Materials & Methods

### 2.1. Study Design

In this retrospective cohort study, we evaluated the medical records of all consecutive female patients who underwent laparoscopic gynecologic surgery under general anesthesia between 1 January 2015 and 30 April 2021. Ethical approval for this study (registration no. 2021-07-038) was obtained from the relevant institutional review board.

### 2.2. Patient Selection

A mix of menstruating and postmenopausal female patients aged 19–65 years who underwent elective laparoscopic gynecologic surgery (laparoscopic subtotal hysterectomy; laparoscopically assisted vaginal hysterectomy; or total laparoscopic radical hysterectomy, including bilateral salpingectomy, bilateral salpingo-oophorectomy, or lymphadenectomy) under general anesthesia were considered eligible for inclusion in this study.

We excluded patients who were on any medications or had an implanted device that could affect the cardiovascular function, heat balance, or levels of the reproductive hormones, as also those who had a history of thyroid disease, dysautonomia, Raynaud’s syndrome, diabetes mellitus, or hypertension. Patients with an anesthesia duration of <1 h or those converted to open surgery were also excluded.

The patients were divided into two groups according to the occurrence of inadvertent intraoperative hypothermia: the non-hypothermia group (*N* = 472) and hypothermia group (*N* = 188). After propensity score matching, 312 patients (*N* = 156 in each group) were analyzed.

### 2.3. Perioperative Management

The general anesthesia procedure for all laparoscopic gynecologic surgeries was per the routine protocol at our hospital. General anesthesia was induced with 2 mg/kg of 1% propofol and 0.6 mg/kg of 1% rocuronium. Mechanical ventilation using the volume-controlled mode of the Primus anesthesia workstation (Dräger, Lübeck, Germany) was commenced with a tidal volume of 8 mL/kg and a frequency of 12 breaths/min. The respiratory rate was adjusted to maintain an end-tidal carbon dioxide pressure of approximately 35 mm Hg. Desflurane concentration during the maintenance phase of anesthesia was titrated to maintain a bispectral index value of 40–60 by adjusting the vaporizer setting at ±1.0 vol%. Electrocardiogram, heart rate, non-invasive blood pressure, peripheral oxygen saturation, end-tidal concentrations of oxygen, carbon dioxide, and desflurane, and body temperature were monitored.

After the induction of general anesthesia, a nasopharyngeal temperature probe (D-2336-2011; Dräger Medical GmbH, Lübeck, Germany) was inserted through the nasal cavity, and a 9.5 to 10.0 cm depth was set for optimal placement. The nasopharyngeal temperature was recorded every 10 min until the end of surgery. Ambient temperature was maintained at 21 °C ± 3 °C inside the operating room at all times with corresponding relative humidity between 20 to 60%. Intravenous fluids at room temperature, warmed irrigating fluid and heating and humidifying peritoneal insufflation gas (CO_2_) were administered during surgery.

All patients received a forced-air warming blanket (Bair Hugger^®^, model 750; Arizant Healthcare, Minneapolis, MN, USA), which delivered forced air at 43 °C, from before induction of anesthesia until the end of surgery.

On arrival in the post-anesthesia care unit (PACU), the patients’ body temperature was measured using an infrared tympanic membrane (TM) thermometer (Braun ThermoScan, IRT 4520; Braun GmbH, Kronberg, Germany). In patients with a TM temperature <36 °C, an active warming device was used to warm the patient’s body until discharge from the PACU.

Postanesthetic shivering was defined as a visible and involuntary muscular activity in the face, jaw, head, trunk, or extremities. Patients who experienced shivering that lasted for >10 min were administered 25 mg meperidine intravenously.

### 2.4. Primary and Secondary Outcomes

The primary outcome was the levels of female reproductive hormones as potential predictors of inadvertent intraoperative hypothermia. The secondary outcomes were the incidence of postanesthetic shivering and treatment in the PACU, the association of inadvertent intraoperative hypothermia with postanesthetic shivering and meperidine use, and the risk factors associated with inadvertent intraoperative hypothermia.

### 2.5. Statistical Analysis

Statistical analyses were performed using SPSS (version 25.0; SPSS Inc., Chicago, IL, USA). The patient groups were compared using an independent t-test or Mann–Whitney U test according to the result of the normality test for continuous variables. Categorical variables were analyzed using the chi-square test. The association of inadvertent intraoperative hypothermia with the levels of female reproductive hormones was analyzed using the Phi (φ) coefficient. The association of inadvertent intraoperative hypothermia with postanesthetic shivering and meperidine use was analyzed using the point-biserial correlation coefficient. Binary logistic regression analysis was performed to identify the risk factors associated with inadvertent intraoperative hypothermia. Predictor variables were assessed to ensure that they met the linearity assumption of logistic regression, and those that did not satisfy the assumption were categorized. For model selection, candidate predictors with a univariate association of *p* < 0.05 were identified using a stepwise forward selection procedure. Thereafter, we assessed the models for problems with collinearity and examined the key variables for effect modification using interaction terms.

Variables that differed between the two groups were subjected to propensity score matching, which resulted in balanced variables between the non-hypothermia and hypothermia groups at *p* > 0.1 and a standardized mean difference (SMD) of <0.1. Variables such as age, BMI, fresh gas flow rate, and duration of anesthesia that could influence the occurrence of inadvertent intraoperative hypothermia were selected for propensity score matching, and the SMDs of ordinal variables were calculated as nominal variables. No further adjustments were made after confirming a balance between the two groups as a criterion of *p* > 0.1 and SMD < 0.1.

Data are presented as mean ± standard deviation, median or number (%) of patients. Odds ratios were calculated with 95% confidence intervals (CI). For all analyses, statistical significance was set at *p* < 0.05.

## 3. Results

A total of 1120 patients underwent laparoscopic gynecologic surgery at our hospital between 1 January 2015 and 30 April 2021. Among them, 460 patients were excluded because of incomplete medical records or due to the exclusion criteria. Thus, 660 patients were included in this study. Propensity score matching for baseline and clinical characteristics identified 312 paired patients in the two groups (*N* = 156 per group).

No significant differences were observed between the non-hypothermia and hypothermia groups in terms of age, menstrual cycle phase, or proportion of postmenopausal women before and after matching. However, after propensity score matching, the incidence of hypothermia was significantly lower in patients in the luteal phase of the menstrual cycle than in those in the follicular phase or in postmenopausal patients (*p* < 0.01). Body mass index (BMI) was significantly lower in the hypothermia group than in the non-hypothermia group before (*p* < 0.01) and after matching (*p* < 0.01). The nasopharyngeal temperature before anesthesia induction and at anesthesia reversal was significantly lower in the hypothermia group than in the non-hypothermia group both before (*p* < 0.01) and after matching (*p* < 0.01). The estradiol levels before and after matching (*p* < 0.01) and the progesterone levels before (*p* < 0.01) and after matching (*p* < 0.05) were significantly lower in the hypothermia group than in the non-hypothermia group. Fresh air at high flow rate (3 or 4 L/min) was significantly more frequent in the hypothermia group than in the non-hypothermia group before and after matching. The duration of surgery and anesthesia was significantly longer, and the amount of fluid infused was significantly higher in the hypothermia group than in the non-hypothermia group both before (*p* < 0.01) and after matching (*p* < 0.01) (Table 1).

The tympanic core body temperature, incidence of postanesthetic shivering, and frequency of meperidine use in the PACU were significantly lower in the hypothermia group than in the non-hypothermia group, both before (*p* < 0.01) and after matching (*p* < 0.01) (Table 2).

The association of estradiol levels (r = −0.218, *p* = 0.000) and progesterone levels (r = −0.235, *p* = 0.000) with inadvertent intraoperative hypothermia was significant but weakly negative before matching. However, the correlation of estradiol (r = −0.326, *p* = 0.000) and progesterone (r = −0.485, *p* = 0.000) levels with inadvertent intraoperative hypothermia was significant and moderately negative after matching (Table 3).

The correlation between inadvertent intraoperative hypothermia and the incidence of postanesthetic shivering was significant (*p* = 0.000) and weakly positive (φ = 0.287). The correlation between inadvertent intraoperative hypothermia and the frequency of meperidine used was significant (*p* = 0.000) and moderately positive (φφ = 0.468) (Table 4).

In binary logistic analysis, the odds ratio for estradiol was 0.995 (*p* = 0.014, 0.993 < CI < 0.998) before matching and 0.993 (*p* = 0.000, 0.862 < CI < 0.930) after matching, and that for progesterone was 0.895 (*p* = 0.000, 0.862 < CI < 0.930) before matching and 0.833 (*p* = 0.014, 0.990 < CI < 0.996) after matching. The odds ratio for BMI was 0.538 (*p* = 0.000, 0.481 < CI < 0.601) before matching and 0.508 (*p* = 0.000, 0.435 < CI < 0.93) after matching. The odds ratio for nasopharyngeal temperature before the anesthesia induction was 0.002 (*p* = 0.000, 0.001 < CI < 0.010) before matching and 0.002 (*p* = 0.000, 0.001 < CI < 0.007) after matching. The odds ratio for fresh air flow rate was 2.458 (*p* = 0.000, 1.736 < CI < 3.482) before matching and 10.117 (*p* = 0.000, 6.001 < CI < 17.056) after matching. The odds ratio for the duration of anesthesia was 1.364 (*p* = 0.000, 1.282 < CI < 1.451) before matching and 1.133 (*p* = 0.000, 1.094 < CI < 1.175) after matching (Table 5 and Table 6).

## 4. Discussion

The main finding of this study was that the levels of female reproductive hormones might influence the occurrence of inadvertent intraoperative hypothermia. The association of estradiol and progesterone levels with inadvertent intraoperative hypothermia was significant and moderately negative. The odds ratios for the levels of female reproductive hormones as predictors of inadvertent intraoperative hypothermia were significant but close to 1.

Many studies have reported the effects of female reproductive hormones or the menstrual cycle on thermoregulation in healthy people or in patients undergoing surgeries under general anesthesia [7,8,9,10]. Previous studies reported that the core body temperature remained unchanged under cold exposure during the follicular and luteal phases; however, it was consistently higher in the luteal phase than in the follicular phase [7,9,10]. The core body temperature under general anesthesia was higher in the luteal phase, but it decreased significantly in both follicular and luteal phases at anesthesia reversal [8].

In the present study, the incidence of hypothermia in patients in the luteal phase was significantly lower than in those in the follicular phase or post menopause. The estradiol and progesterone levels were significantly lower in the hypothermia group than in the non-hypothermia group. Therefore, estradiol and progesterone levels were associated with inadvertent intraoperative hypothermia. However, the odds ratios for the levels of female reproductive hormones were close to 1, indicating that they might not be risk factors for inadvertent intraoperative hypothermia during general anesthesia.

Individual differences are seen in the normal body temperature depending on physiological factors such as age, circadian rhythm, metabolic factors, and phases of the menstrual cycle [11]. The potential factors that can influence the occurrence of intraoperative hypothermia include ambient temperature, BMI, duration of anesthesia, flow rate of fresh air, type of anesthesia, age, sex, hemodynamic status, and preoperative body temperature [6]. At present, there is no strong evidence implicating any single independent variable as a causative factor that increases the risk of intraoperative hypothermia.

The present study showed that low nasopharyngeal temperature before anesthesia induction, low BMI, high flow rate of fresh air, and longer duration of anesthesia might be the causative factors that increase the risk of inadvertent intraoperative hypothermia.

The findings of this study were based on using a forced-air warming blanket in all patients. The incidence of postanesthetic shivering in hypothermic patients in the PACU was 34%, which was consistent with that reported in a previous study [5].

The present study differs from a previous study [8] on female reproductive hormones. The previous study reported only the effects of the phases of the menstrual cycle on thermoregulation under general anesthesia. The present study demonstrated the effects of the levels of female reproductive hormones on inadvertent intraoperative hypothermia during gynecologic surgery under general anesthesia.

The present study has several limitations. First, the retrospective cohort design might have introduced selection and information biases [12]. Moreover, this study had a small sample size, which limits the generalizability of the results. Studies with larger sample sizes might be required to clarify controversial outcomes. Second, we could not identify whether positioning the probe on the mucosa of the upper or midportion of the nasopharynx was the optimal choice. Suboptimally placed nasopharyngeal temperature probes could show large differences in the measured core body temperature compared to that with optimally placed probes [13]. Third, although we excluded patients whose reproductive hormone levels did not match each phase of the menstrual cycle, individual variance in reproductive hormone levels throughout the menstrual cycle and information bias might have affected the identification of the early, mid, or late stage in each phase of the menstrual cycle. To understand this relationship, prospective studies that can control various biases are required. Finally, we could not confirm whether all patients in the PACU whose core body temperature was measured using an infrared TM thermometer were examined to exclude ear debris. The aural readings in this study might have been affected by ear debris and the measurement of ear canal temperature [14].

## 5. Conclusions

Estradiol and progesterone levels were associated with inadvertent intraoperative hypothermia. However, the odds ratio for female reproductive hormone levels was close to 1. Therefore, female reproductive hormones may not be a risk factor for hypothermia during gynecologic surgery under general anesthesia. However, the small sample size in this study limits the generalizability of the results. Further studies are needed to clarify the relationship of reproductive hormones and intraoperative hypothermia.

## Figures and Tables

**Table 1:** Demographic data.

Variables	Before Matching			After Matching		
	Non-Hypothermia (*N* = 472)	Hypothermia (*N* = 188)	*p*-Value	Non-Hypothermia (*N* = 156)	Hypothermia (*N* = 156)	*p*-Value
Age (years)	48.2 ± 5.5	48.4 ± 5.8	0.720	47.9 ± 5.2	48.6 ± 5.8	0.263
Body mass index (kg/m^2^)	24.3 ± 2.0	21.9 ± 1.4	0.000	24.4 ± 1.9	22.1 ± 1.6	0.000
Nasopharyngeal body temperature before anesthesia induction (°C)	37.1 ± 0.2	36.9 ± 0.2	0.000	37.1 ± 0.2	36.9 ± 0.2	0.000
Estradiol (pg)	97.0 ± 81.9	74.3 ± 68.4	0.000	104.5 ± 78.6	67.3 ± 65.6	0.021
Progesterone (ng)	5.5 ± 6.7	2.5 ± 3.8	0.00	8.6 ± 8.1	1.9 ± 3.1	0.001
Phases of the menstrual cycle			0.114			0.000
Follicular	149 (31.6)	81 (43.1)		36 (23.1)	72 (46.2)	
Luteal	184 (39.0)	48 (25.5)		79 (50.6)	33 (21.2)	
Menopause	139 (29.4)	59 (31.4)		41(26.3)	51 (32.7)	
Fresh gas flow rate			0.002			0.001
Low (≤1 L/min)	280 (59.3)	70 (37.2)		123 (78.8)	42 (26.9)	
High (3 or 4 L/min)	192 (40.7)	118 (62.8)		33 (21.2)	114 (73.1)	
Type of surgery			0.000			0.000
Laparoscopic subtotal hysterectomy	126 (26.7)	18 (9.6)		27 (28.7)	10 (10.6)	
Laparoscopically assisted vaginal hysterectomy	344 (72.9)	54 (28.7)		66 (70.2)	29 (30.9)	
Total laparoscopic radical hysterectomy	2 (0.4)	116 (61.7)		1 (1.1)	55 (58.5)	
Duration of anesthesia (min)	77.0 ± 5.6	127.4 ± 29.2	0.000	78.3 ± 13.9	126.2 ± 31.2	0.000
Duration of surgery (min)	47.0 ± 5.7	97.4 ± 29.2	0.000	48.4 ± 14.4	96.3 ± 31.3	0.000
Amount of fluid infused (mL)	827.3 ± 184.4	1305.8 ± 461.6	0.000	829.5 ± 185.2	1302 ± 460.3	0.000
Nasopharyngeal body temperature at anesthesia reversal (°C)	36.2 ± 0.1	35.5 ± 0.2	0.000	36.1 ± 0.1	35.5 ± 0.3	0.000

Values are expressed as mean ± standard deviation or numbers (%).

**Table 2:** Postanesthetic shivering and treatment in the PACU.

Variables	Before Matching			After Matching		
	Non-Hypothermia (*N* = 472)	Hypothermia (*N* = 188)	*p*-Value	Non-Hypothermia (*N* = 156)	Hypothermia (*N* = 156)	*p*-Value
TM temperature (°C)	36.1 ± 0.1	35.4 ± 0.2	0.000	36.2 ± 0.1	35.4 ± 0.2	0.000
Postanesthetic shivering	48 (10.2)	64 (34.0)	0.000	12 (7.7)	53 (34.0)	0.000
Meperidine (25 mg) use	8 (1.7)	64 (34.0)	0.000	2 (1.3)	53 (34)	0.000

Values are expressed as mean ± standard deviation or number (%). TM, tympanic membrane; PACU, post-anesthesia care unit.

**Table 3:** Association of inadvertent intraoperative hypothermia with female reproductive hormones throughout the menstrual cycle.

	Variables	Before Matching			After Matching		
		Inadvertent Hypothermia	Point-Biserial Coefficient	*p*-Value	Inadvertent Hypothermia	Point-Biserial Coefficient	*p*-Value
Phase of the menstrual cycle	Estradiol		−0.218	0.000		−0.326	0.000
	Progesterone		−0.235	0.000		−0.485	0.000

**Table 4:** Association of inadvertent intraoperative hypothermia with postanesthetic shivering and meperidine use.

Variables	Before Matching			After Matching		
	Inadvertent Hypothermia	Phi (φ) Coefficient	*p*-Value	Inadvertent Hypothermia	Phi (φ) Coefficient	*p*-Value
Postanesthetic shivering		0.287	0.000		0.324	0.000
Meperidine (25 mg) use		0.468	0.000		0.429	0.000

**Table 5:** Binary logistic regression analysis of risk factors for inadvertent intraoperative hypothermia before matching.

Variables	B	Exp(B)	*p*-Value	95% CI
Age	0.010	0.639	0.613	0.978 < CI < 1.039
Body mass index	−0.620	0.538	0.000	0.481 < CI < 0.601
Nasopharyngeal body temperature before anesthesia induction	−6.204	0.002	0.000	0.001 < CI < 0.007
Estradiol	−0.005	0.995	0.014	0.993 < CI < 0.998
Progesterone	−0.110	0.895	0.000	0.862 < CI < 0.930
Phase of the menstrual cycle	−0.149	0.62	0.167	0.697 < CI < 1.064
Flow rate of fresh air	0.899	2.458	0.000	1.736 < CI < 3.482
Duration of anesthesia	0.310	1.364	0.000	1.282 < CI < 1.451

CI, confidence interval.

**Table 6:** Binary logistic regression analysis of risk factors for inadvertent intraoperative hypothermia after matching.

Variables	B	Exp(B)	*p*-Value	95% CI
Age	0.023	1.023	0.262	0.983 < CI < 1.066
Body mass index	−0.677	0.508	0.000	0.435 < CI < 0.593
Nasopharyngeal body temperature before anesthesia induction	−6.156	0.002	0.000	0.001 < CI < 0.010
Estradiol	−0.007	0.993	0.014	0.990 < CI < 0.996
Progesterone	−0.183	0.833	0.00	0.793 < CI < 0.875
Phase of the menstrual cycle	−0.263	0.769	0.069	0.581 < CI < 1.018
Flow rate of fresh air	2.314	10.117	0.000	6.001 < CI < 17.056
Duration of anesthesia	0.125	1.133	0.000	1.094 < CI < 1.175

CI, confidence interval.
